# No, it is not a third breast that grows!

**DOI:** 10.11604/pamj.2018.30.213.16402

**Published:** 2018-07-16

**Authors:** Erdogan Nohuz, Marie-Claire Bui

**Affiliations:** 1 Clermont-Auvergne University, Place Henri Dunant Clermont-Ferrand and General Hospitals of Thiers Route du Fau Thiers, France; 2 General Practitioner Office Henri IV 45, Boulevard Henri IV, Ambert France

**Keywords:** Dermatofibrosarcoma protuberans, soft tissue sarcoma, sarcomatous transformation, recurrence, aggressive cutaneous tumo

## Image in medicine

Dermatofibrosarcoma protuberans (DFSP) is a rare slow growing skin neoplasm which is characterized by a low potential of malignancy. It rarely shows metastasis to local lymph nodes or distant sites. However, it appears locally aggressive with a tendency to peripheral tumor extensions and recurrence. While DFSP can occur in all ages, it affects in the majority of cases the patients between the third and the fifth decades of age. A high index of suspicion should be taken when facing unusual lesion with a history of slow but persistent growth. DFSP involves most commonly the trunk and extremities but can also be located at any site such as scalp, head, neck, breast, pubic area and vulva. Here, we present a case of recurrent DFSP in a 52-year-old woman with a slow enlarging mass in the upper of the thorax, between the two breasts. She was previously managed by surgery several years before for a DFSP developed at the same site. Complete resection including wide surgical margins of at least 3 cm followed by immediate reconstruction is the treatment of choice to minimize recurrence rate. Marked propensity of DFSP to recur necessitates referral to a specialized center to achieve optimal oncological resection and avoid unnecessary re-operation. However, the minimum resection margin required remains undefined. Radiation and chemotherapy have been tried with limited success, although adjuvant radiotherapy may be used in advanced cases where surgery is no longer feasible. Imatinib can contribute to disease control in patients with locally advanced or metastatic DFSP.

**Figure 1:**
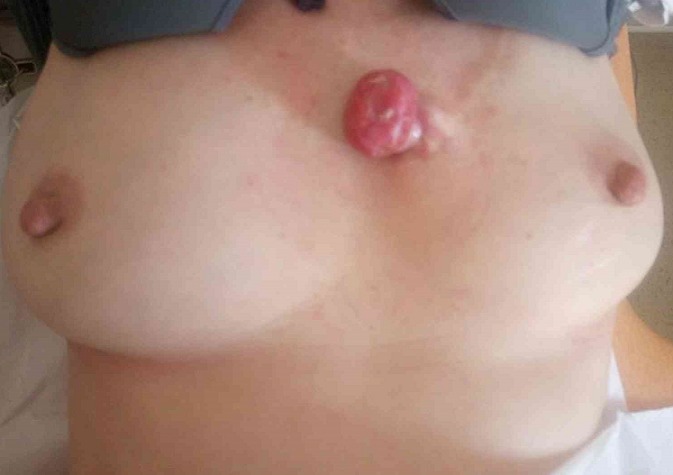
Tumor protruded above the surface of the skin located at the medial upper part of the thorax, between the two breasts

